# A bibliometric and visual analysis of publications on artificial intelligence in colorectal cancer (2002-2022)

**DOI:** 10.3389/fonc.2023.1077539

**Published:** 2023-02-07

**Authors:** Pan Huang, Zongfeng Feng, Xufeng Shu, Ahao Wu, Zhonghao Wang, Tengcheng Hu, Yi Cao, Yi Tu, Zhengrong Li

**Affiliations:** ^1^ Department of General Surgery, First Affiliated Hospital of Nanchang University, Nanchang, China; ^2^ Department of Digestive Surgery, Digestive Disease Hospital, The First Affiliated Hospital of Nanchang University, Nanchang, China; ^3^ Medical Innovation Center, The First Affiliated Hospital of Nanchang University, Nanchang, China; ^4^ Department of Pathology, The First Affiliated Hospital of Nanchang University, Nanchang, China

**Keywords:** artificial intelligence, deep learning, colorectal cancer, bibliometrics, visualization, CiteSpace, VOSviewer

## Abstract

**Background:**

Colorectal cancer (CRC) has the third-highest incidence and second-highest mortality rate of all cancers worldwide. Early diagnosis and screening of CRC have been the focus of research in this field. With the continuous development of artificial intelligence (AI) technology, AI has advantages in many aspects of CRC, such as adenoma screening, genetic testing, and prediction of tumor metastasis.

**Objective:**

This study uses bibliometrics to analyze research in AI in CRC, summarize the field’s history and current status of research, and predict future research directions.

**Method:**

We searched the SCIE database for all literature on CRC and AI. The documents span the period 2002-2022. we used bibliometrics to analyze the data of these papers, such as authors, countries, institutions, and references. Co-authorship, co-citation, and co-occurrence analysis were the main methods of analysis. Citespace, VOSviewer, and SCImago Graphica were used to visualize the results.

**Result:**

This study selected 1,531 articles on AI in CRC. China has published a maximum number of 580 such articles in this field. The U.S. had the most quality publications, boasting an average citation per article of 46.13. Mori Y and Ding K were the two authors with the highest number of articles. *Scientific Reports*, *Cancers*, and *Frontiers in Oncology* are this field’s most widely published journals. Institutions from China occupy the top 9 positions among the most published institutions. We found that research on AI in this field mainly focuses on colonoscopy-assisted diagnosis, imaging histology, and pathology examination.

**Conclusion:**

AI in CRC is currently in the development stage with good prospects. AI is currently widely used in colonoscopy, imageomics, and pathology. However, the scope of AI applications is still limited, and there is a lack of inter-institutional collaboration. The pervasiveness of AI technology is the main direction of future housing development in this field.

## Introduction

Colorectal cancer (CRC) is currently the third most prevalent and the second most deadly cancer worldwide. As many countries’ economies continue to grow, the incidence of CRC will increase (1, 2). In addition, the incidence of CRC is trending younger (3, 4).

Due to the increasing incidence of CRC, early screening and diagnosis of CRC are particularly important. Polyps cause most CRCs. This process begins with an aberrant crypt and progresses through 10-15 years, eventually leading to CRC (5). Colonoscopy with pathology biopsy is the standard for diagnosing CRC. However, there are still some limitations to endoscopic biopsy. The level of the endoscopist directly affects the detection rate of adenomas. Less experienced physicians can miss up to 50% of adenomas compared to skilled physicians (6).

Artificial Intelligence (AI) is a new technological science for research and development to simulate human intelligence. There are two main branches of AI in medicine: virtual and physical (7). Machine learning is a representation of the virtual part. It uses a large amount of existing data for algorithmic analysis to form a specialized logic set. This logic allows us to make judgments on new data (8). Imaging omics and predictive models belong to this category of applications. Another application of AI is mainly the application of physical devices. A typical example is various intelligent robotic systems, such as Da Vinci Robot-assisted Surgical Systems and intelligent care robots (9, 10). A study by Chen et al. (11) on applying deep neural network technology to colonoscopy showed that the system’s accuracy was significantly better than that of general practitioners in screening for tumors and polyps. This study reveals the significant advantages of AI in information recognition. In the past five years, AI has been widely used to diagnose (12) and treat (13–15) CRC.

While the current use of AI in various aspects of CRC has yielded surprising results, we cannot ignore some of its disadvantages (16, 17). For example, AI can only train and build neural networks for a single task and cannot handle multiple tasks. AI also has significant limitations in treating rare diseases (18). In addition, considerable differences remain in the sensitivity, specificity, and accuracy of AI in CRC (19). Therefore, more randomized controlled studies are needed for further validation to improve the effectiveness and specificity of AI systems.

AI in CRC field is currently in the early stages of development. On average, more than 300 relevant studies are published each year, with the number continuing to grow. It has become a challenge for many scholars to keep abreast of the research and future trends. Bibliometrics is the discipline of quantitative analysis of literature using mathematical and statistical methods. Due to the rigor and objectivity of bibliometrics, scholars in many fields use this method to study the corresponding fields (20). We can use bibliometrics to analyze authors, journals, keywords, references, citations, and other information in specific databases to understand the current research structure and collaboration patterns in a field and to predict future research trends (21). Bibliometrics is now widely used in many fields (22–26). Our team has also researched the clinical applications of AI (27). However, as of now, there are no bibliometric studies related to AI in CRC.

Therefore, we hope to analyze the research process and status of research in the past 20 years and predict the possible future research trends by collecting the relevant literature on AI in the field of CRC from relevant databases. This study will help scholars in the area have a more systematic understanding of the research priorities and future research trends.

## Method

### Data source

Our data are from the Science Citation Index Expanded (SCI-EXPANDED) of the Web of Science Core Collection. Web of Science (WOS) is an extensive, comprehensive, multidisciplinary, core journal citation database containing more than 15,000 leading, high-impact journals and 50,000,000 publications in 251 categories and 150 research areas (28). Each article integrates the year, country and region, abstract, author, institution, document type, research field, journal title, citations, and references (29). Many scholars consider databases to be the most suitable for literature analysis.

### Search strategy

**Figure 1:**
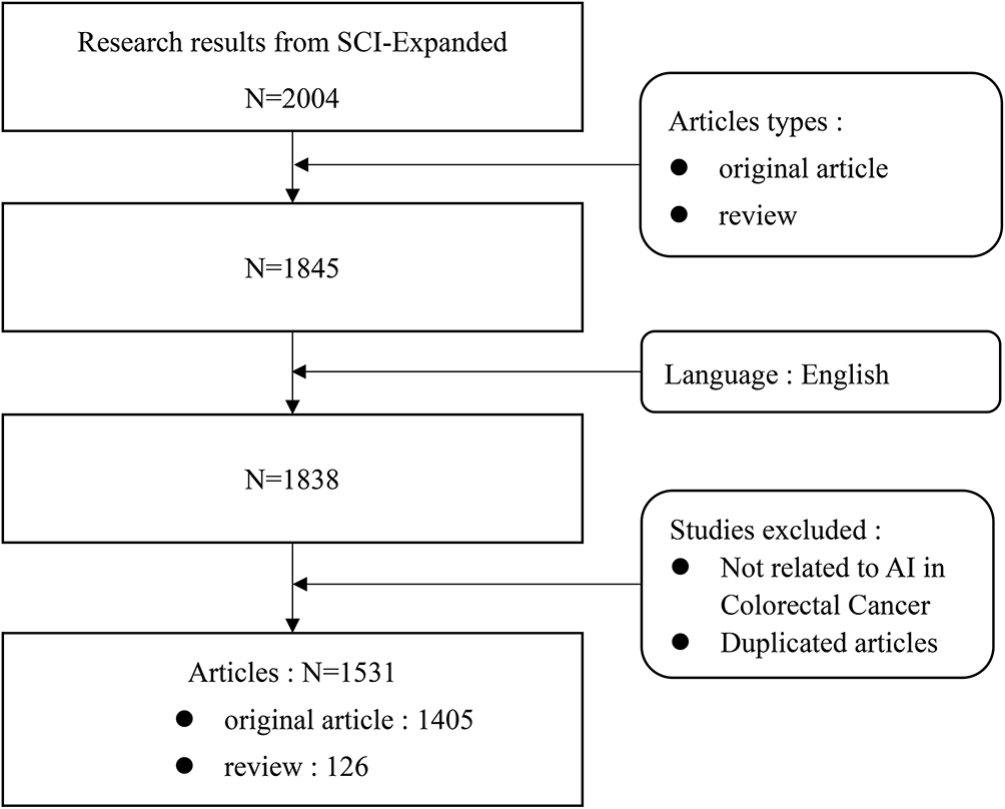
Flowchart of literature screening.

We searched and collected literature related to AI in the field of CRC from January 1st, 2002, to September 30th, 2022. The type of literature was limited to Articles and Reviews, and the language was limited to English. We searched and screened all the papers within one day to ensure the consistency of the data. The data was exported to the WOS website as “full record and cited references” in “plain text format.” **Figure 1** shows the screening process.

The search formula is in the **Supplemental File**.

### Data analysis and visualization

We conducted a bibliometric analysis of the documents retrieved. The main items analyzed were countries and regions, authors, institutions, citations and references, journals, and cited journals. Two investigators completed data analysis and checked independently to ensure study accuracy and reproducibility.

The H-index refers to a scholar with at most H papers cited at least H times each. Because it considers production and influence while resisting the bias of highly cited articles, it accurately reflects a scholar’s scholarly achievements (30, 31). Impact factor (IF) is widely used to evaluate the impact of journals and is a simple yet effective indicator (32). We use the 2021 edition of Journal Citation Reports (JCR) and IF to assess the value of journals (33). The Altmetric Attention Score (AAS) is a new metric for assessing the impact of articles (34). It uses weighted algorithms to collect data from various origins, including news, Twitter, Google, Facebook, personal blogs, and other social media. It analyzes that data to demonstrate the impact of an article (35). The AAS can be accessed through a free search site (https://www.scienceopen.com/).

We used Microsoft Excel 2019 for flowcharts and statistical tables. We used the free statistics website (https://bibliometric.com/) and SCImago Graphica 1.0.25 for analysis and graphing of country and regional postings and collaborative postings. This study uses Citespace 6.1.R3 and VOSviewer 1.6.18 for the bibliometric analysis of countries, authors, journals, institutions, keywords, references, and citations. The primary analysis methods include co-authorship, co-citation, and co-occurrence, which are common in bibliometrics.

CiteSpace is a JAVA-based visualization software that allows visualization and analysis of academic literature in the research field. The analysis includes keywords, authors, journals, countries (36, 37).

VOSviewer is also a visualization software for bibliometric literature analysis, with similar functionality to Citespace (38). Compared to Citespace, VOSviewer’s clustering analysis is more intuitive and aesthetically pleasing, and it can export data to SCImago Graphica for geographic visualization.

### Ethics statement

The data used in this study were acquired from an open source and did not require approval by any ethical committee.

## Result

### Global publishing and collaboration trends

Following the literature search strategy flowchart, we collected 1531 papers from SCI-Expanded (SCI-E) over the past 21 years, including 1405 treatises and 126 reviews. These papers were published in 520 journals by 9126 authors from 2523 institutions in 77 countries. The articles cited 48,166 documents from 8794 journals.

**Figure 2:**
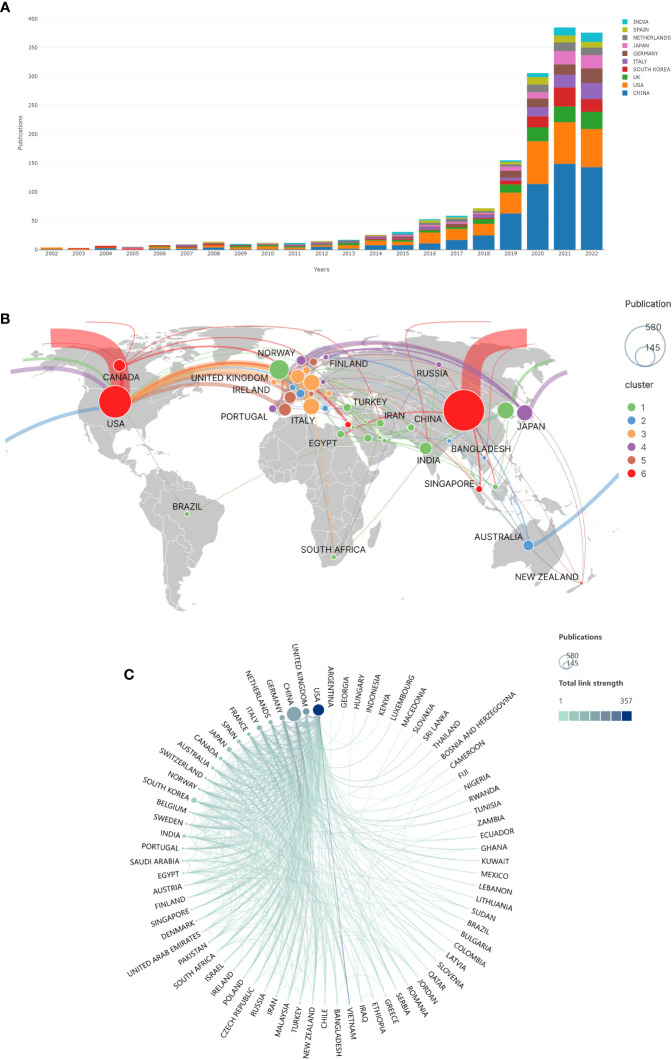
Publications and cooperation in different countries/regions of the world. **(A)** Top 10 countries/regions with annual publication trends of AI in CRC. **(B)** Map of the world’s countries/regions in terms of publications and collaborations in the field of AI in CRC. (The size of the circle represents the number of articles issued. The thickness of the connecting line represents the number of collaborative communications between countries. The color of the circles represents the intensity of cooperation. Countries with the same color cooperate more frequently with each other.). **(C)** Cooperation between countries/regions (The size of the circle represents the number of articles issued, and the thickness of the line represents the intensity of cooperation between countries/regions.).

**Figure 2A** shows that the number of articles issued each year gradually increases. Especially after 2019, the number of publications has multiplied. Among them, the papers published in 2020-2022 were over 300, 379 in 2021, and 354 in 2022 (9 months of data).

### Bibliometric analysis of countries

The world map (**Figure 2B**) shows the volume of publications in each country in AI in CRC. As seen from the figure, research in this field is mainly concentrated in East Asia, North America, and Western Europe. The volume of papers is hugely unevenly distributed among countries.

The most published articles were by Chinese scholars (**Table 1**). They issued a total of 580 pieces, accounting for 37.9% of published articles, but the average citations for their papers were 16.06, which was at a medium level. It was followed by the US and the UK, with 361 and 136 articles, respectively. Only three countries have more than 100 articles, ten countries have more than 50, and the remaining countries have fewer articles. The most citations per article were in the United States, with 361 papers cited 16,653 times and 46.13 citations per article.

**Table 1:** Top 7 productive countries/regions related to AI in CRC.

Rank	Country	Publication	Citation	Publication/Citation
1	China	580	9317	16.06
2	USA	361	16653	46.13
3	UK	136	4006	29.46
4	South Korea	97	1089	11.23
5	Italy	95	1384	14.57
6	Germany	92	2345	25.49
7	Japan	87	1233	14.17

**Figure 2C** depicts the cooperation between countries. The US has the most comprehensive collaboration with other nations, including China, the UK, and Germany. The US, the UK, China, Germany, and the Netherlands collaborate the most in issuing articles. These head countries cooperate more closely, while other countries have weak cooperation.

### Bibliometric analysis of authors

**Figure 3:**
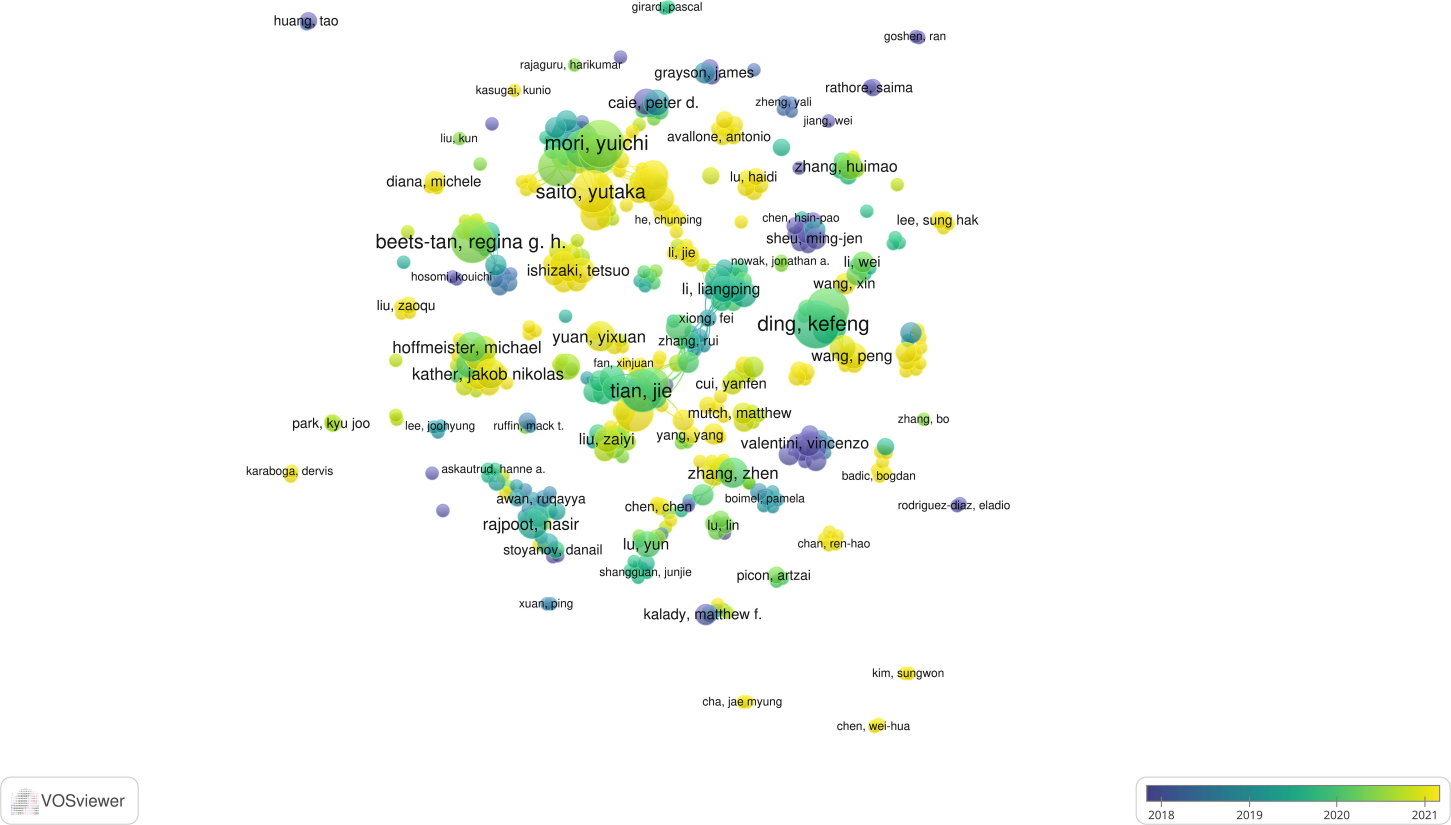
Visualization of co-authorship through VOSviewer (Circles represent the number of articles published. Connecting lines represent collaboration between authors. Colors represent the average year of authors’ publications.).

We can understand the representative scholars and core strength of research in this field through the co-authorship analysis of the authors. We can calculate the minimum number of articles published by core authors in this field by Price’s Law: $n=0.749\times \sqrt{n_{max}}=2.48$ (*n* is the minimum number of papers published by core authors, and *n*_max_ is the maximum number of documents published by a single author in the field). Therefore, we import the data of authors with more than three articles into VOSviewer for visualization, and we can obtain the co-authorship visualization graph (**Figure 3**). As seen from the figure, there is a lack of collaboration between most authors. National scholars dominate collaboration among authors, and stable partnerships among international ones have not been formed.

We have listed the top 10 authors with the most published articles (**Table 2**). Among the 10 authors, Japanese scholars were the most numerous (5), followed by Chinese scholars (4) and Dutch scholars (1). The most published articles were by Japanese scholar Yuichi Mori and Chinese scholar Kefeng Ding. In the field of AI in CRC, Yuichi Mori has published 11 articles with 290 citations and an average citation count of 26.36. He collaborated closely with Shin-Ei Kudo, Masashi Misawa, Kensaku Mori and other authors. The most numerous citation is by Chinese scholar Jie Tian, who has published ten papers in this field with a record of 1552 citations, with an average of 155.20 per paper. He also has the highest H-index, much higher than other scholars.

**Table 2:** Top 10 authors by publications.

Rank	Author	Country	Count	Total citations	Average Citation	H-index
1	Yuichi Mori	Japan	11	290	26.36	29
2	Kefeng Ding	China	11	65	5.91	21
3	Jie Tian	China	10	1552	155.20	76
4	Regina G H Beets-Tan	Netherlands	10	247	24.70	12
5	Yutaka Saito	Japan	10	186	18.60	38
6	Jun Li	China	10	53	5.30	20
7	Masashi Misawa	Japan	9	283	31.44	26
8	Shin-Ei Kudo	Japan	9	239	26.56	19
9	Kensaku Mori	Japan	9	215	23.89	36
10	Zhenhui Li	China	9	80	8.89	6

### Analysis of journals and cited journals

A total of 520 journals published articles in this field, of which 74 journals published more than five articles. Twenty-eight journals published more than ten articles. We list the top 10 journals with the most publications in **Table 3**. The top 3 most published journals were *Scientific Reports* (51,3.33%), *Cancers* (46,3.00%), and *Frontiers in Oncology* (46,3.00%). Among the top 10 journals, the most cited journal was *Scientific Reports*, with 1,215 citations and an average citation rate of 23.82.

**Table 3:** Top 10 most published journals in AI in CRC.

Rank	Journal	IF (2021)	JCR(2021)	Publication	Citation	Average Citation/Publication
1	Scientific Reports	4.996	Q2	51	1215	23.82
2	Cancers	6.575	Q1	46	265	5.76
3	Frontiers in Oncology	5.738	Q2	46	153	3.33
4	PloS One	3.752	Q2	26	331	12.73
5	IEEE Access	3.476	Q2	24	170	7.08
6	World Journal of Gastroenterology	5.374	Q2	21	209	9.95
7	Applied Sciences-basel	2.838	Q2	19	116	6.11
8	Computer Methods and Programs in Biomedicine	7.027	Q1	18	285	15.83
9	Computers in Biology and Medicine	6.698	Q1	17	302	17.76
10	Diagnostics	3.992	Q2	16	68	4.25

**Figure 4:**
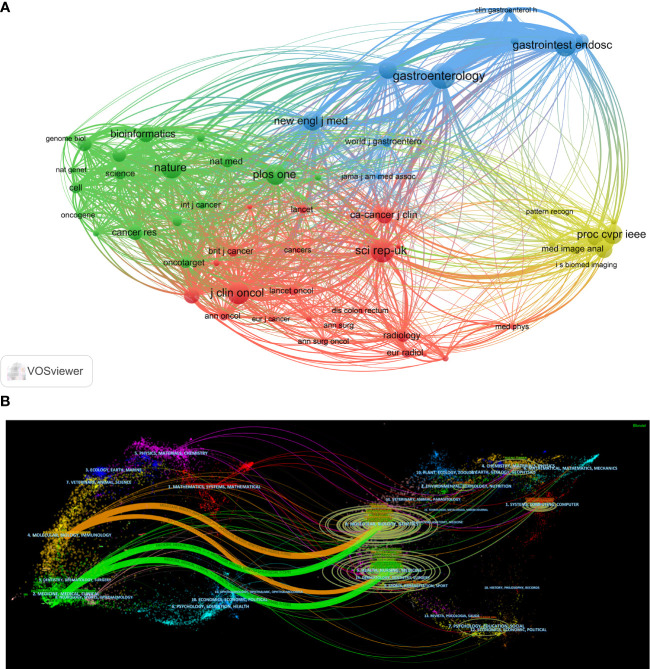
Citation relationship between journals. **(A)** Co-citation relationships between journals (The circles represent the number of articles cited by CRC in a journal, and the connecting lines represent a paper citing two different journals separately). **(B)** A dual-map overlap of journals on AI in CRC (On the left side are the citing journals. On the right side are the cited journals. The color represents the classification of journals. The curve is the citation line. The ellipse’s long axis represents the number of papers cited in the same subject journal. The short axis of the ellipse represents the number of authors of papers in journals on the same topic.).

All papers cited references in a total of 8794 journals. We imported journal data with more than 200 citations into VOSviewer for visual analysis to obtain the co-citation web of cited journals (**Figure 4A**). The top three most-cited journals were *Gastroenterology* (1117 citations), *Scientific Reports* (1037 citations), and *Gastrointestinal Endoscopy* (951 citations). The cited journals consisted of four different color clusters. The green clusters are mainly for journals in Basic areas such as cell biology and molecular biology. The reason for citing these journals is to review the current research results and to provide theoretical support for their research. The blue and red clusters are clinically oriented journals in the field of gastrointestinal tumors. The yellow areas are journals in the field of computer science. Research often cites these journals to provide technical support.

We use Citespace to visualize the citing relations between citing and cited journals (**Figure 4B**). In the field of AI in CRC, there are 3 main areas of citing journals: (1) Medicine, Medical, Clinical; (2) Molecular, Biology, Immunology; (3) Mathematics, Systems, Mathematical. The cited journals are mainly in 6 fields: (1) Health, Nursing, Medicine; (2) Molecular, Biology, Genetics; (3) Systems, Computing, Computer; (4) Chemistry, Materia, Physics; (5) Psychology, Education, Social; (6) Environmental, Toxicology, Nutrition.

### Analysis of research institutions

**Figure 5:**
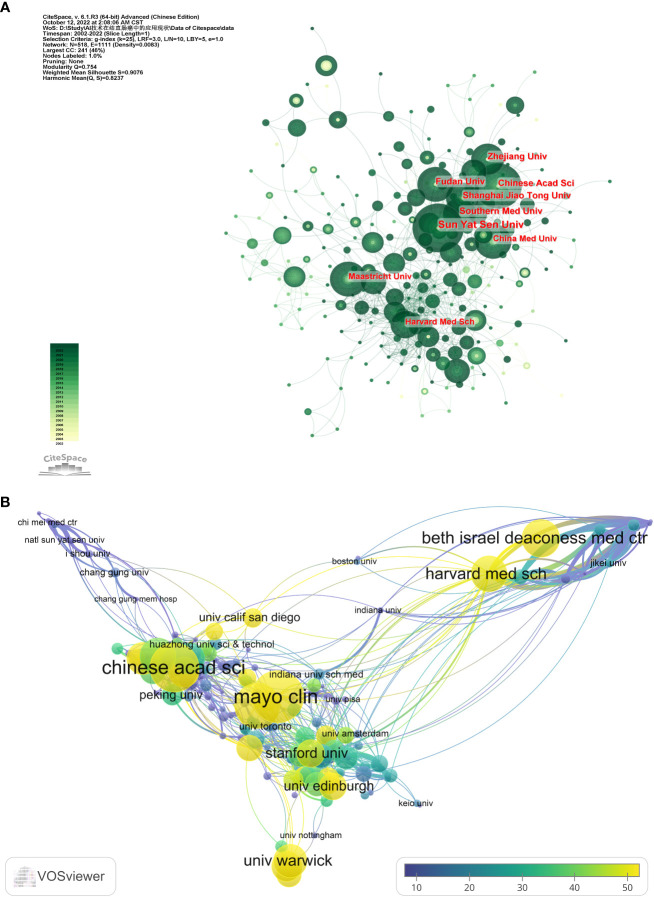
Cooperation and citations between institutions. (The circle size represents the number of articles issued by the institution. Connecting lines represent the intensity of collaboration between institutions.). **(A)** Cooperation among institutions. **(B)** Average citations per article by different institutions.

In AI in CRC, 2523 institutions have researched and published papers on the subject (**Figure 5A**). Of these, only 58 institutions published more than ten papers, and 186 institutions published more than five papers. We list the ten institutions with the highest publications and visualize institutional collaborations and citations.

The top three institutions with an enormous number of paper outputs were Sun Yat-sen University (51), Chinese Academy of Sciences (33), and Shanghai Jiao Tong University (33) (**Table 4**). The most cited institutions were, in order, Chinese Academy of Sciences (2047), Harvard Medical School (1212), and Southern Medical University (1212), which are also the three most cited institutions in terms of average citations (**Figure 5B**). Except for these large institutions, there is no gap in the number of articles published by most institutions. There is more cooperation between institutions within each country compared to the lack of cooperation between most inter-country institutions.

**Table 4:** Top 10 institutions with publications in AI in CRC.

Rank	Institution	Publication	Citation	Average Citation/Publication
1	Sun Yat Sen University	51	898	17.61
2	Chinese Academy of Sciences	33	2047	62.03
3	Shanghai Jiaotong University	33	311	9.42
4	Southern Medical University	30	1212	40.4
5	Zhejiang University	30	306	10.2
6	Fudan University	30	973	32.43
7	Maastricht University	26	649	24.96
8	Harvard Medical School	25	1212	48.48
9	China medical university	24	150	6.25
10	University of Oslo	21	626	29.81

### Co-occurrence analysis of keywords

**Figure 6:**
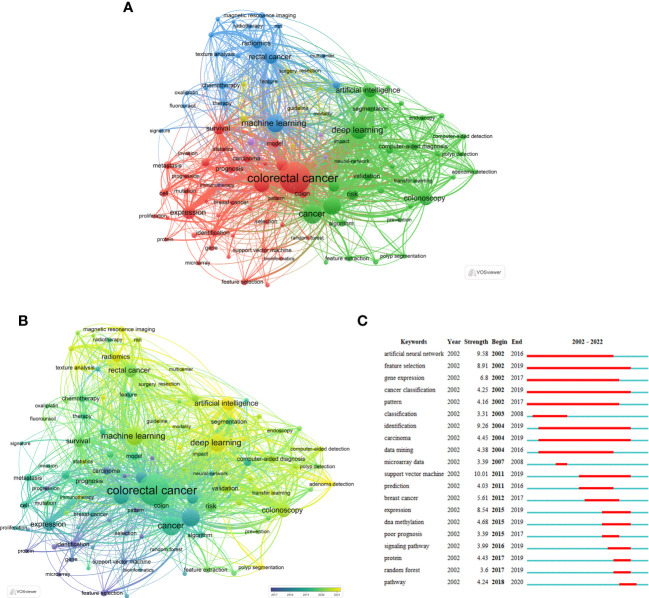
Co-occurrence analysis of keywords (The node size represents the frequency of keywords, the line between nodes represents two keywords appearing in the same document at the same time.). **(A)** Clustering view of keywords co-occurrence analysis (The node color represents keyword clustering.). **(B)** Temporal view of keywords co-occurrence analysis (The node color represents the average year of keyword occurrence.). **(C)** The top 20 burst words.

We extracted keywords from these documents for analysis. The sum of keywords in 1531 papers was 5203, among which 107 keywords appeared more than 20 times. Keywords such as colorectal cancer (562), classification (233), machine learning (233), and deep learning (223) appear most frequently. We import the keywords with more than 20 frequencies into VOSviewer for visualization (**Figure 6**).

These keywords can be roughly divided into four categories (**Figure 6A**). The keywords in red are clustered around CRC and include secondary keywords such as expression, survival, feature selection, biomarker, and other secondary keywords. It is mainly about the training and recognition of CRC-related biometric features by AI technology, which belongs to basic research. The keywords of green clustering are mainly around Deep Learning, Computer-aided Diagnosis, Colonoscopy, and other keywords, which are mainly about classification, auxiliary diagnosis, and treatment of colonoscopic tumors. The blue and purple clusters have Machine Learning, Chemotherapy, and Radiomics as secondary keywords, mainly focusing on imaging and pathological examination of CRC. The yellow sets have fewer high-frequency keywords, such as Surgery and Resection, which are primarily related to the application of AI in the surgical treatment of CRC.

**Figure 6B** shows the average year of keyword appearances. As can be seen from the figure: Identification, Feature Selection, and other keywords appeared earlier, mainly before 2018, while Deep Learning, Artificial Intelligence, radiomics, and different keywords appeared more often after 2021. This picture also indicates that the hot research topics in the last few years have concentrated on deep learning, colonoscopy, polyp segmentation, and radiomics.

If some keywords are concentrated in a certain period, we can call them to burst words. Burst words can reflect different stages of development in a field. We extracted the top 20 most breaking keywords from AI papers in CRC by Citespace (**Figure 6C**). AI in CRC first emerged in 2002. After 2015, the duration of burst words gradually shortened. The keyword with the highest burst intensity is the support vector machine.

### Analysis of articles and references

We screened 1531 publications from the field, 41 of which were quoted over 100 times. We presented the top 10 publications with total citations (**Table 5**). Guyon et al. (39) carried out a project on the application of support vector machines in gene selection, which received 5486 citations, much higher than other articles. Tajbakhsh et al. (40) and Huang et al. (41) followed, receiving 1379 and 928 citations, respectively. In the meantime, Wang et al. (42) and Urban et al. (43) have received many citations in AI in CRC. The AAS of Caravagna et al. (44) and Wang et al. (42) were much higher than the rest of the publications.

**Table 5:** Top 10 most cited articles.

Title	Journal	Author	Year	Citation	AAS
Gene selection for cancer classification using support vector machines	Machine learning	Guyon I; et al	2002	5486	27
Convolutional Neural Networks for Medical Image Analysis: Full Training or Fine Tuning?	IEEE Transactions on Medical Imaging	Tajbakhsh N; et al	2016	1379	41
Development and Validation of a Radiomics Nomogram for Preoperative Prediction of Lymph Node Metastasis in Colorectal Cancer	Journal of Clinical Oncology	Huang, YQ; et al	2016	928	4
A Colorectal Cancer Classification System That Associates Cellular Phenotype And Responses to Therapy	Nature Medicine	Sadanandam A; et al	2013	660	76
Locality Sensitive Deep Learning for Detection and Classification of Nuclei in Routine Colon Cancer Histology Images	IEEE Transactions on Medical Imaging	Sirinukunwattana K; et al	2016	595	15
Detecting Repeated Cancer Evolution from Multi-Region Tumor Sequencing Data	Nature Methods	Caravagna G; et al	2018	474	629
Deep Learning Localizes and Identifies Polyps in Real Time With 96% Accuracy in Screening Colonoscopy	Gastroenterology	Urban G; et al	2018	309	58
Real-Time Automatic Detection System Increases Colonoscopic Polyp and Adenoma Detection Rates: A Prospective Randomized Controlled Study	GUT	Wang P; et al	2019	294	594
Gene Expression Patterns Unveil A New Level of Molecular Heterogeneity in Colorectal Cancer	Journal of Pathology	Budinska E; et al	2013	274	24
The Applications of Radiomics in Precision Diagnosis and Treatment of Oncology: Opportunities and Challenges	Theranostics	Liu ZY; et al	2019	272	1

**Figure 7:**
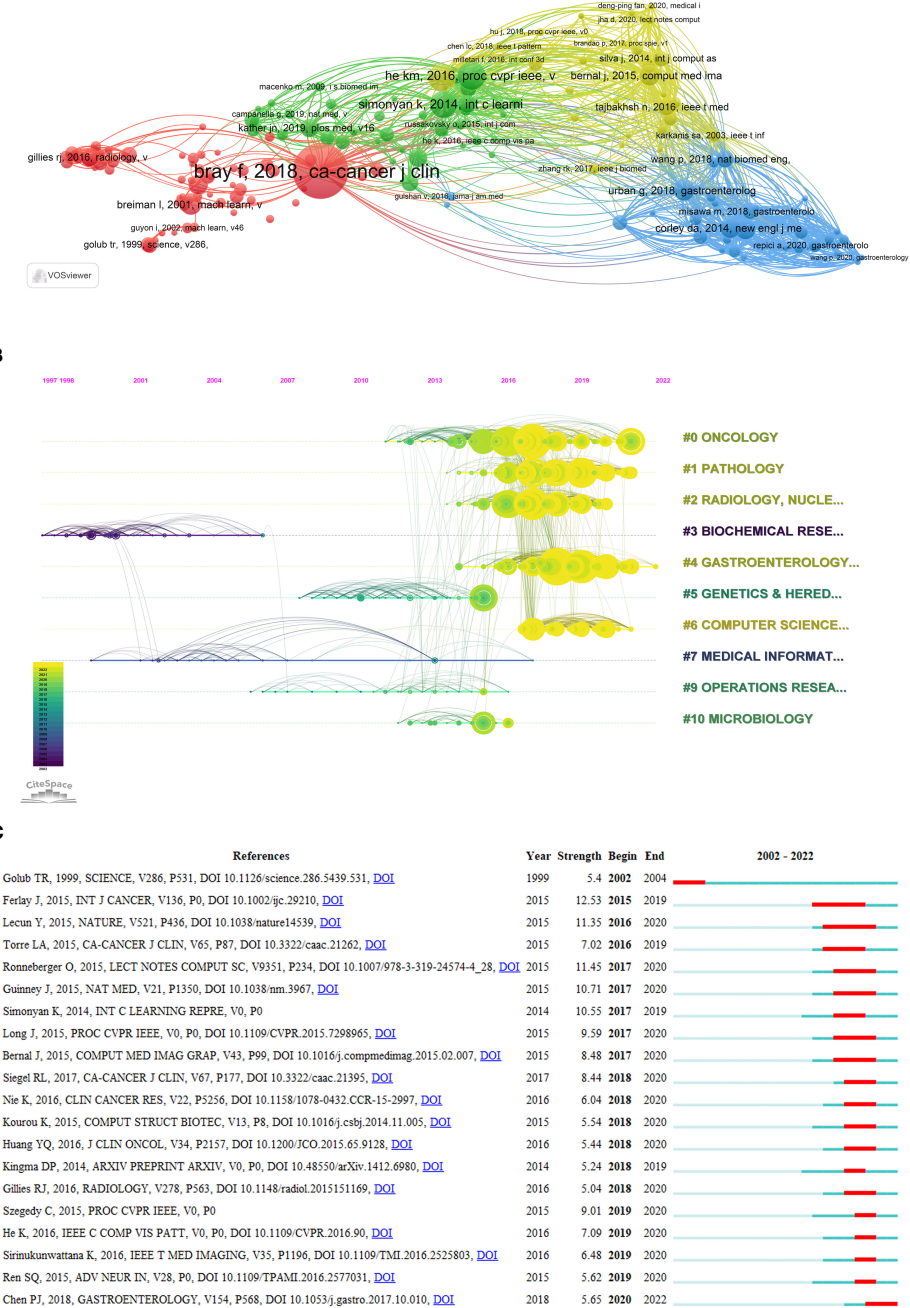
Analysis of reference citations (The circle represents the number of citations. The line represents two articles cited by the same article.). **(A)** Co-citation analysis of references (The colors represent the clustering of references.). **(B)** Timeline diagram of references (The color represents the average time the reference was cited.). **(C)** Top 20 references cited in burst.

All articles cited 48166 references, 161 of which were quoted at least 20 times. We import them with more than 20 citations into VOSviewer for co-citation analysis and visualization (**Figure 7A**). The concerns are divided into four main clusters: articles in the green and yellow clusters are mainly related to computers and AI, and the references specifically provide technical support. The red and blue collections focus on specific applications of AI in CRC, where the red is mainly in imaging histology and pathology, and the blue is mainly in colonoscopy. **Table 6** contains the top 10 references with the most citations. The most extensively cited article is Bray et al. (45), with 186 citations, which focused on the epidemiological data of cancer. The following most cited articles were He et al. (46) and Ronneberger et al. (47), with 95 and 91 citations, respectively. In addition, these ten references, except Bray et al. (45), can be divided into two categories, one for theoretical studies of AI and one for studies of AI applications in clinical settings.

**Table 6:** Top 10 references with the most citations.

Title	Journal	Author	Year	Citation	AAS
Global cancer statistics 2018: GLOBOCAN estimates of incidence and mortality worldwide for 36 cancers in 185 countries	CA: A Cancer Journal for Clinicians	Bray F; et al	2018	186	2454
Deep Residual Learning for Image Recognition	IEEE Conference on Computer Vision and Pattern Recognition	He KM; et al	2016	95	688
U-Net: Convolutional Networks for Biomedical Image Segmentation	Lecture Notes in Computer Science	Ronneberger O; et al	2015	91	263
Very Deep Convolutional Networks for Large-Scale Image Recognition	arXiv 2014	Simonyan K; et al	2014	90	313
Deep Learning Localizes and Identifies Polyps in Real Time With 96% Accuracy in Screening Colonoscopy	Gastroenterology	Urban G; et al	2018	72	58
Random Forests	Machine Learning	Breiman L; et al	2001	71	155
Adenoma Detection Rate and Risk of Colorectal Cancer and Death	New England Journal Of Medicine	Corley DA	2014	70	701
WM-DOVA maps for accurate polyp highlighting in colonoscopy: Validation *vs*. saliency maps from physicians	Computerized Medical Imaging And Graphics	Bernal J; et al	2015	69	3
Real-time automatic detection system increases colonoscopic polyp and adenoma detection rates: a prospective randomized controlled study	GUT	Wang P; et al	2019	69	594
The consensus molecular subtypes of colorectal cancer	Nature Medicine	Guinney J; et al	2015	64	535

We can visualize the classification and publication time of the references by the timeline map (**Figure 7B**). Most of the literature was published after 2016 from the four categories of Oncology, Pathology, Radiology, and Gastroenterology. There were fewer co-citations of references between the different categories in the earlier period.

**Figure 7C** shows the references that were burst cited, and it is clear that there was a spike in burst cited references after 2016, indicating that the field of AI in CRC started to develop rapidly after 2016. The reference with the most burst strength is Ferlay et al. (48), who investigated the global epidemiology of cancer in 2012.

## Discussion

AI technology has been evolving rapidly since its emergence and has been applied in several disciplines. The application of AI in CRC started in 2002 (39). Bibliometrics allows analysis of authors, institutions, countries, and references in SCIE literature databases to understand a research area and visualize it through Citespace and VOSviewer. This research approach is more comprehensive in analyzing the literature and presenting more intuitive results than a general systematic review. In AI in CRC, this research first uses bibliometrics to explore the applications and developments in the area from 2002 to 2022 and to speculate on future research trends.

AI in CRC research was slow to develop until 2015, with fewer than 30 publications per year, and a gradual rise began in 2016. After 2019, more than 100 papers are published each year and growing at a rate of more than 100 papers per year. The documents are expected to exceed 400 in 2022 (**Figure 2A**). This phenomenon indicates that the field is rapidly growing at the moment. The top three countries in this field published more than 1000 articles, accounting for more than 70% of publications from all nations. This result reveals a significant research gap between countries worldwide in this field, with the head country having a decisive advantage over the others. The overall amounts of articles contributed by Chinese scholars were 580. Still, the average number of citations per article is low, 16.06 per article, similar to other Asian countries such as Japan, Korea, and India. However, the average citations are still a gap between China and the Occident, suggesting that the quality of papers from China still has a particular hole compared with that from Europe & US. By digging deeper into the data, we found that China’s annual publication volume begins to surpass that of the United States only after 2018 and will be twice as high by 2022. The average publication date for Chinese scholars is August 2019, compared to May 2018 for the US, which suggests that China is a late starter in this field but is developing rapidly, which may be one of the reasons for the low average citations. The US produced the second most published articles, with 361 in total, which received a staggering 16,653 citations, with an average citation rate of 46.13. It suggests that the US is at the core of this sector. **Figures 2B, C** show the collaborations among different nations. There is a substantial amount of cooperation between China and the US. Germany and the Netherlands, Italy, and other European countries cooperate closely. It shows that the cooperation between countries tends to be regionalized, such as Central Asia and West Asia cooperating more strongly, while European countries and cooperation are close too. However, the cooperation between regions is less, and language may be one of the reasons for this phenomenon.

Co-authorship analysis lets one learn about the collaborative relationships between authors in a discipline. **Figure 3** shows that there is a lack of collaboration among most scholars. However, the extensive range of co-authorship networks among Japanese scholars suggests that cooperation between Japanese scholars is frequent. **Table 2** contains the top 10 authors with the most papers, five of which are Japanese scholars. The most prolific author is Yuichi Mori, who has published 11 articles, and ten co-authored with four other scholars. The situation is similar for other Japanese scholars, which is the chief factor in the large percentage of Japanese scholars in the table. Yuichi Mori’s main research interests are the implementation of AI in colonoscopy, including increasing the diagnosis rate of colonoscopy through AI (49, 50) and predicting the effect of endoscopic tumor removal through AI (51, 52). Jie Tian is the most cited author among the ten authors, with a much higher average citation of 155.20. His H-index of 76 also proves that he is an influential scholar in this area of research. He focuses on the application of AI in histology and pathology imaging. Tian J et al. developed a radiomic columnar map for predicting lymph node metastasis in CRC preoperatively in 2016 (41). This paper has received 930 citations. Currently, he continues to delve into imaging histology, including training the AI to evaluate pathological response to neoadjuvant chemotherapy for rectal cancer *via* MRI (53–55). A survey of the impact of articles in this field (number of citations, AAS) gives us an idea of the critical academic results that have been achieved in this field (**Table 5**). Caravagna et al. (44) and Wang et al. (42) obtained 629 and 594 AAS, respectively, which were much higher than other scholars. The reason is that these two articles were retweeted several times on Twitter.

Articles in AI in CRC are published in a relatively scattered number of journals, with only 28 publishing more than ten articles. The top ten journals by publications are all excellent journals with JAR Q2 or above. Among them, *Scientific Reports* published 51 papers. They received 1215 citations, with an average of 23.82 citations, which is much higher than other journals, indicating that this journal has a significant influence in the field of AI in CRC. The top three journals were *Scientific Reports*, *Cancer*, and *Frontiers in Oncology*, with over 40 articles, much higher than the rest of the journals, indicating that these were more focused on research in this area. Scholars in this field can prioritize their results for publication in these journals. In addition, *Computer Methods and Programs in Biomedicine*, *Computers in Biology*, and *Medicine* have very high citations per article and are also excellent journals. These two journals mainly focus on computer principles (56–58), while Articles published in other journals focused on clinical applications. AI in CRC is an interdisciplinary field. The primary references in the published papers are from 6 areas, which indicates that the collaboration between fields is widespread and the field’s future development will require closer collaboration between disciplines.

China accounts for 7 of the top 10 institutions, while the US, the Netherlands, and Norway each have one. China has a substantial amount of research institutions and publications, mainly due to the strong support of the Chinese government for AI applications in recent years, which has happened in almost all areas involving AI (59–63). It is foreseeable that, with increasing investment, China may be a leader in this field of research in the future. Each article published by the Chinese Academy of Sciences, Southern Medical University, and Harvard Medical School received more than 40 citations, indicating that they are the central institutions within the field.

The analysis of keywords provides another perspective on the development process and trends in this field. **Figure 6A** demonstrates that the keywords in AI in CRC can be divided into 4 clusters and combined with the period of the keywords in **Figures 6B, C**. We can divide the development of this field into two stages. The first stage is before 2018, mainly with red clustering keywords, such as Biomarker, Expression, Feature Selection, and Support Vector Machine. This is the technology reserve period, and scholars from various countries mainly conducted theoretical research on AI and the development of some basic applications. Guyon et al. (39) applied a Support Vector Machine to gene selection, and Chen et al. (64) and Lee et al. (65) improved the Support Vector Machine. On the other hand, Xu et al. (66) attempted to apply weakly supervised learning to classify pathological images. Keywords with blue and green clusters frequently appeared after 2018, such as Computer-aided Diagnosis, Machine Learning, Radiomics, and Colonoscopy, indicating that related research is starting to develop toward clinical applications. Urban et al. (43) applied CNN to colonoscopic adenoma screening, and Wang P et al. conducted several prospective studies on AI-assisted detection of adenomas (42, 67), all with satisfactory results. Several Meta-analyses (68, 69) have also confirmed the great advantage of AI technology in endoscopic adenoma detection. The application of AI in pathological examination has mainly focused on the identification of slides by AI assistance. Echle et al. (70) developed a deep learning-based system that directly detects CRC MSI by HE-stained slides. Yamashita et al. (71) also conducted a related study and showed that AI performed far better than experienced gastrointestinal pathologists. In addition, CT- or MRI-based imaging histology has many applications, including assessing pathological responses after radiotherapy or chemotherapy (72) and predicting colon cancer infiltration and metastasis (73, 74). The duration of keyword bursts was long before 2016 and became shorter after 2016 (**Figure 6C**). This phenomenon indicates that AI in CRC developed slowly before 2016 and entered a rapid development stage after 2016. The short burst duration due to the accelerated technology iteration may cause the inability to detect the outbreak of words in the line after 2020.

The analysis of co-cited references can reflect the reasons for the development of this field. Most of the highly cited references (46, 47) are from the field of computing (**Table 6**). It suggested that the development of AI technologies dominates the development of the field. There is still an explosion of citations, suggesting that this field is in a phase of rapid development.

In general, the application of AI in CRC can be divided into two phases. The first stage is 2002-2018, mainly involving the accumulation of AI technologies, and many scholars have conducted preliminary trials in this field. The second phase started from 2018 to the present. In this stage, AI technologies are beginning to apply to clinical applications, and the leading applications fall into three directions. The first category is the application in colonoscopy. Urban et al. (43) applied a convolutional neural network (CNN) to colonoscopy to improve the adenoma detection rate. The results showed that the accuracy of CNN in identifying polyps was 96.4%. Wang et al. (42) compared the real-time automatic polyp detection system with standard colonoscopy. They showed that the number of smaller adenomas detected by the AI system was much higher than that of the conventional examination (185 *vs*. 102). Repici et al. (75) reported similar results in their study. The second type of application is the application in imaging examinations. Lu et al. (76) applied R-CNN to MRI to predict lymph node metastasis and showed that the diagnosis time of AI was only 1/30 of that of imaging physicians. Cusumano et al. (77) developed a field-strength independent MR radiomics model to predict the achievement of pCR after neoadjuvant chemotherapy for rectal cancer and also achieved good results. The third type of application is in the pathology of CRC. Digital pathology (DP) can be used to obtain high-quality full-slide pathology image data by computer to form digital or virtual sections. AI powered by deep learning can process these medical images rapidly in a standardized manner and help pathologists improve their diagnostic efficiency and reduce their workload by outlining and rendering suspicious images in a structured language. Xu et al. (78) proposed a deep neural network-based method to classify, segment, and visualize large histopathological images. In the segmentation of malignant tissue glands, this method achieved 98% accuracy. Yamashita et al. (71) developed a deep learning model (MSINet) to predict microsatellite instability (MSI) in CRC. The results of external validation performed by the AI-trained model showed that the area under the receiver operating characteristic curve (AUROC) amounted to 0.865 (95% CI 0.735-0.995), while the average performance of the AUROC of the five pathologists was 0-605 (95% CI 0.453-0.757). The above study demonstrates the potential of AI deep learning applied to digital pathology to improve the quality and efficiency of pathology diagnosis significantly.

AI technology still has some shortcomings, and data is still the core part of AI. Deep learning of AI requires hugely high data quality, and data collection is challenging and expensive because of privacy and security issues. Secondly, AI technology currently builds models that only apply to a specific clinical range and become inapplicable once they go beyond that range. These limitations make it difficult for one AI model to be universally applicable worldwide. The security of AI system data is also an important issue that needs to be resolved. In addition, deep learning models often seem more like “black boxes,” which are end-to-end learning designs that absorb data and generate output conclusions without explicitly explaining the rationale and process for their output conclusions (79). Therefore, the future development of AI in CRC may focus on the following two aspects. First, as globalization progresses, deep learning algorithms can train and learn using globally shared data and build an AI disease prediction model for patients worldwide. Second, the future AI can break the model bias directly through the most essential, fundamental features to build a model, quantify the features, explain the process of AI results, and solve the current “black box” problem.

## Limitation

There are still some flaws in this study. First, the field’s most recent and high-quality articles may be overlooked due to insufficient citations. Second, research is limited to English literature, and critical studies in other languages may be missed. What is more, research in the literature may have a certain lag in the current state-of-the-art research, which may bias the prediction of future directions.

## Conclusion

Currently, AI has been widely used in the treatment of CRC. The main applications of AI today are in 3 areas. First, it is used in colonoscopy to improve the detection rate of adenomas and tumors at colonoscopy. The next is pathology, which can help pathologists identify pathological sections more quickly and accurately. The final is the application in imaging histology, mainly to predict the degree of infiltration and metastasis and to evaluate the efficacy of radiotherapy and chemotherapy. China and the United States are leading in this field, and the gap with other countries is still widening. Cooperation between most countries is still lacking. The future development of this field will largely depend on the availability of more significant accounts and more data sources for AI deep learning to improve its generalizability.

## Data availability statement

The original contributions presented in the study are included in the article/**Supplementary Material**. Further inquiries can be directed to the corresponding authors.

## Author contributions

PH and ZF conceived the study; ZW and TH collected the data; XS and AW checked and analyzed the data; PH and ZF wrote the paper; and YC, YT, and ZL reviewed and revised the paper. All authors contributed to the article and approved the submitted version.
