# Supplementary material for: A bibliometric and visual analysis of publications on artificial intelligence in colorectal cancer (2002-2022)
Source: Front Oncol. 2023 Feb 7;13:1077539. doi: 10.3389/fonc.2023.1077539 (PMC9941644; doi:10.3389/fonc.2023.1077539)
Supplement: Supplementary file 1 [file DataSheet_1.docx]

The search formula is : ((TS=(“artificial intelligence” OR “artificial neural network” OR “adversarial generative” OR “active learning” OR “Bayes network” OR “computational intelligence” OR “Convolutional Neural Networks” OR “Cellular Neural Network” OR “continual learning” OR “contrastive learning” OR “deep learning” OR “deep network” OR “deep neural network” OR “data mine” OR “data mining” OR “domain adaptation” OR “expert system” OR “feature extraction” OR “feature learning” OR “feature mining” OR “feature embedding” OR “few-shot learning” OR “feature selection” OR “graph learning” OR “graph mining” OR “intelligent learning” OR “instance segmentation” OR “image segmentation” OR “knowledge graph” OR “meta learning” OR “machine learning” OR “metric learning” OR “neural nets model” OR “neural network” OR “neural learning” OR “reinforcement learning” OR “Semantic segmentation” OR superpixel OR self-supervised OR “supervised learning” OR “semi-supervised” OR “transfer learning” OR “unsupervised learning” OR “unsupervised clustering”)) AND TS=(“Colonic Cancer” OR “Colon Cancer” OR “Cancer of Colon” OR “Cancer of the Colon” OR “Colonic Neoplasm” OR “Colon Neoplasm” OR “Neoplasm of Colon” OR “Neoplasm of the Colon” OR “Colonic Tumor” OR “Colon Tumor” OR “Tumor of Colon” OR “Tumor of the Colon” OR “Rectal Cancer” OR “Rectum Cancer” OR “Cancer of Rectum” OR “Cancer of the Rectum” OR “Rectal Neoplasm” OR “Rectum Neoplasm” OR “Neoplasm of Rectum” OR “Neoplasm of the Rectum” OR “Rectal Tumor” OR “Rectum Tumor” OR “Tumor of Rectum” OR “Tumor of the Rectum” OR “Colorectal Cancer” OR “Colorectum Cancer” OR “Cancer of Colorectum” OR “Cancer of the Colorectum” OR “Colorectal Neoplasm” OR “Colorectum Neoplasm” OR “Neoplasm of Colorectum” OR “Neoplasm of the Colorectum” OR “Colorectal Tumor” OR “Colorectum Tumor” OR “Tumor of Colorectum” OR “Tumor of the Colorectum”)) AND DOP=(2002-01-01/2022-09-30).
